# A model of weight‐based stigma in health care and utilization outcomes: Evidence from the learning health systems network

**DOI:** 10.1002/osp4.553

**Published:** 2021-08-27

**Authors:** Sean M. Phelan, Katherine W. Bauer, David Bradley, Steven M. Bradley, Irina V. Haller, Manpreet S. Mundi, Lila J. Finney Rutten, Darrell R. Schroeder, Kristin Fischer, Ivana Croghan

**Affiliations:** ^1^ Division of Health Care Delivery Research Mayo Clinic Rochester Minnesota USA; ^2^ Robert D. and Patricia E. Kern Center for the Science of Health Care Delivery Mayo Clinic Rochester Minnesota USA; ^3^ Department of Nutritional Sciences University of Michigan School of Public Health Ann Arbor Michigan USA; ^4^ The Ohio State Wexner Medical Center Columbus Ohio USA; ^5^ Minneapolis Heart Institute and Minneapolis Heart Institute Foundation Minneapolis Minnesota USA; ^6^ Essentia Institute of Rural Health, Essentia Health Duluth Minnesota USA; ^7^ Division of Endocrinology, Diabetes, Metabolism, and Nutrition Mayo Clinic Rochester Minnesota USA; ^8^ Department of Quantitative Health Sciences Mayo Clinic Rochester Minnesota USA; ^9^ Department of Medicine Division of General Internal Medicine Mayo Clinic Rochester Minnesota USA

**Keywords:** health care utilization, obesity, obesity bias, patient‐centered care, social stigma

## Abstract

**Objective:**

Obesity is stigmatized and people with obesity report experiencing stigmatizing situations when seeking health care. The implications of these experiences are not well understood. This study tests an indirect effects model of negative care experiences as an intermediate variable between obesity and care avoidance/utilization and switching primary care doctors.

**Methods:**

A survey was completed by 2380 primary care patients in the Learning Health Systems Network (LHSNet) Clinical Data Research Network with a BMI >25 kg/m^2^. Measures included scales assessing stigmatizing situations, perceived patient‐centered communication, perceived respect, having delayed needed care, and having looked for a new primary doctor in the past 12 months. Sequential and serial indirect effects of care experiences and respect in the association between BMI and care utilization outcomes was modeled.

**Results:**

The hypothesized model was supported by findings. The associations between BMI and delaying needed care (OR = 1.06, *p* < 0.001) and attempting to switch primary doctors (OR = 1.02, *p* = 0.04) was mediated by both stigmatizing situations experienced in a health care context and lower patient‐centered communication. Lower perceived respect mediated the association between care experiences and utilization outcomes.

**Conclusions:**

People with higher BMIs may avoid care or switch doctors as a result of stigmatizing experiences and poor communication with doctors. These outcomes may contribute to morbidity in people with obesity if they delay or avoid care for health concerns when symptoms first present.

## 1 INTRODUCTION

There is ample evidence that obesity is a stigmatized trait and that health care providers as well as the general public are biased against people with obesity.^1^, ^2^, ^3^, ^4^, ^5^, ^6^ Weight stigma consists of negative stereotypes and attitudes about people with overweight or obesity, and discrimination that can result. Commonly held stereotypes among health care providers about people with obesity include that they lack willpower and are non‐adherent to medical recommendations, including advice to lose weight.^7^, ^8^, ^9^, ^10^ These biases and stereotypes can negatively affect both the interpersonal and technical quality of health care.^11^, ^12^, ^13^ People with obesity receive less patient‐centered communication,^12^, ^13^, ^14^ with health care providers initiating less rapport,^15^ making fewer attempts to build relationships, and providing less patient education.^16^ Less patient‐centered communication is, in turn, associated with lower patient satisfaction^17^ and adherence,^18^ worse care outcomes,^19^, ^20^, ^21^ and less adoption of healthy lifestyle behaviors.^22^, ^23^ Technical deficits in care observed during interactions between health care providers and people with obesity include reports of health care providers focusing on weight loss while paying inadequate attention to other complaints and issues^24^ and recommending weight loss in place of medications or other therapies for symptoms.^11^

Patients with obesity report feeling judged and treated with less respect^13^, ^25^, ^26^ than other patients, and subsequently trust their health care providers less.^26^ Thus, health care encounters may be experienced as threatening to patients with obesity, possibly leading to avoidance of future health care encounters and delay of needed care.^27^, ^28^, ^29^ Because of this, people with obesity may seek care later in the progression of a disease, compared to people with normal BMIs, and thus may present with more advanced and possibly harder to treat conditions.^30^ This is one factor that may contribute to the excess morbidity and mortality associated with overweight and obesity.^2^, ^31^, ^32^ Studies have found people with obesity are more likely to delay screening for cancer^33^ and other health care visits.^34^ Additionally, people with obesity may be more likely than others to “doctor shop” or seek a regular health care provider with whom they feel comfortable and well‐treated.^35^, ^36^ Thus, people with obesity may be less likely to have a regular doctor, a factor associated with better care continuity and health outcomes,^37^, ^38^ and may be more likely to receive care at drop‐in or urgent care centers, which are less likely to address lifestyle or behavior issues or chronic disease risks.^39^ However, the associations among obesity, care experiences, and care utilization have been primarily examined separately, thus, this study tests a model of care experiences as mediating the relationship between obesity and care avoidance/utilization.

**FIGURE 1:**
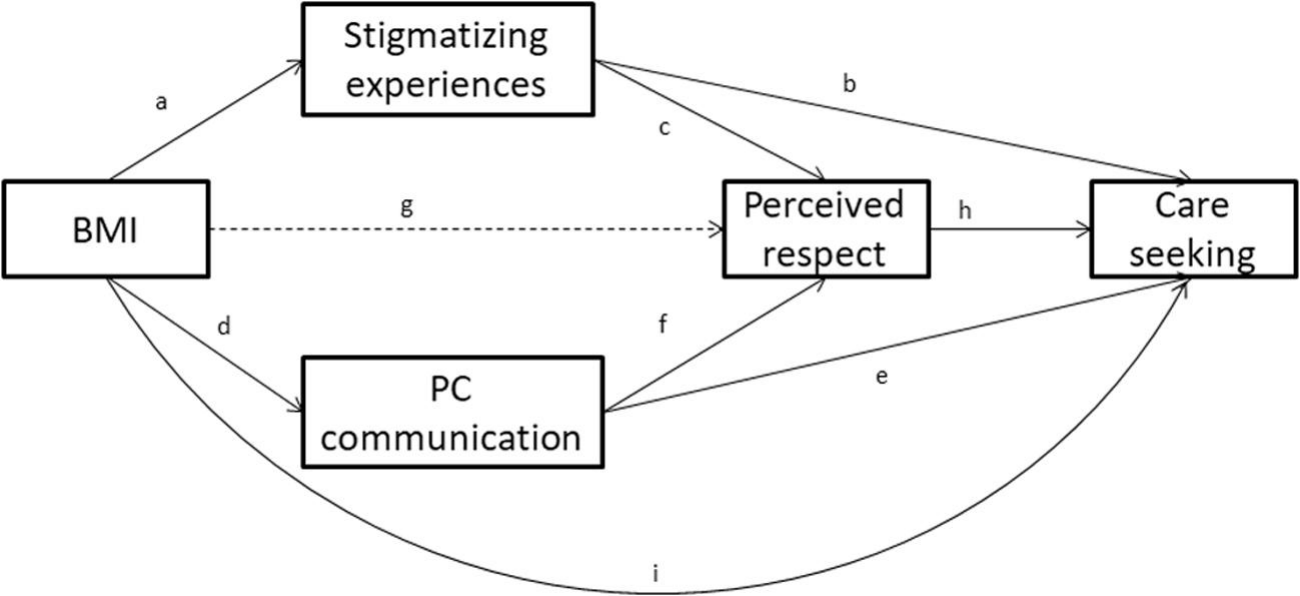
Conceptual model

In the conceptual model (Figure 1), higher BMI is associated with care‐seeking behaviors via two primary pathways. First, (a) higher BMI is associated with more frequent stigmatizing experiences during a health care encounter, such as a health care provider making assumptions about patient behavior based on obesity stereotypes or making embarrassing or cruel remarks, or blaming unrelated symptoms or problems on weight. These experiences may trigger identity threat, or the response to feeling that one's social identity is at risk of being devalued or viewed in terms of a group stereotype.^29^ Social identities are the groups and categories that make up the way people see themselves and believe they are seen by others.^40^ Identity threat causes a stress response that can reduce a person's ability to perform cognitively taxing activities and lead to future avoidance of similar situations.^41^ One's body size is salient in a medical encounter, and people with overweight or obesity who are aware of stereotypes and prejudice against people with larger body sizes may be vigilant for poor treatment or discrimination related to their size.^2^ In response to identity threat, the patient may (b) avoid similar situations in the future, and (c) feel disrespected or humiliated, souring the therapeutic relationship with their health care provider and leading them to (h) avoid that provider and/or seek care elsewhere.

In the second pathway, (d) people with higher BMI experience less patient‐centered communication throughout the health care encounter, which is associated with lower satisfaction with care and (e) may cause patients to seek care elsewhere or avoid follow‐up care, and (f) may engender feelings of disrespect based on health care providers' lower likelihood of building rapport and (h) lead to patients seeking alternate sources of care.

Leveraging a large clinical data research network, the study team examined the relationships between BMI, perceived stigma in health care, patient centered communication, perceived health care provider respect, and two measures of a patient's utilization of health care services related to quality of care: delaying needed care due to weight and trying to find a new primary care provider.

## 2 METHODS

### 2.1 Patient population

This study consisted of a survey of primary care patients in the Learning Health Systems Network (LHSNet) Clinical Data Research Network.^42^ LHSNet consists of data from approximately 10 million patients from nine health systems across the US. Of those health systems, five participated in this survey study of primary care patients with overweight or obesity.^43^ Eligible patients were adults with a health care provider‐documented height and weight corresponding to a BMI >25 kg/m^2^. Pregnant women were excluded if their weight was taken between 6 months prior to and 3 months following a delivery.

LHSNet was developed as a distributed research network wherein each participating site hosts a local collection of data that adheres to the Patient Centered Outcomes Research Network (PCORnet) Common Data Model, including standardized data definitions, format and content across the LHSNet. The distributed data model enables execution of standardized computer algorithms to identify specific patient populations.^43^ The detailed description of the study participants has been described elsewhere.^42^ Using an algorithm to identify obesity and overweight resulted in a cohort of 2,072,572 adult patients with BMI >25 kg/m^2^ across sites.^42^ Each site randomly selected 4000 patients from strata based on BMI with 1000 patients in each category (overweight (BMI, 25–29.9 kg/m^2^), obesity class I (BMI, 30–34.9 kg/m^2^), obesity class II (BMI, 35–39.9 kg/m^2^), obesity class III (BMI, >40 kg/m^2^). From the 20,000 selected patients, 19,964 had valid address information available in the database and were mailed anonymous surveys. Among mailed surveys, 313 were returned as undeliverable; 2799 (14.2%) as completed, and 1033 (5.2%) with refusals to participate. Among completed surveys 2380 had sociodemographic data and reported current height and weight corresponding to BMI ≥ 25 kg/m^2^. Across the sites, between 12.9% and 15.2% of mailed surveys were returned completed. Surveys were anonymous and were mailed once. Surveys were collected during the last two months of 2017 and the first 3 months of 2018.

### 2.2 Survey measures

The survey was developed to measure aspects of the health care experience and health‐related behaviors of patients with overweight and obesity.^42^, ^44^ The survey was designed to be low‐burden and took, on average, 11 min to complete. Measured constructs are described below.

Stigmatizing experiences were measured using a modified Medical Subscale of the Stigmatizing Situations Index.^24^ This subscale measures how frequently the respondent experiences each of four stigmatizing situations while seeking health care, including, “Having a doctor make cruel remarks, ridicule you, or call you names,” and “A doctor blaming unrelated physical problems on your weight.” Participants responded on a 4‐point scale of “Never”, “Once in your life”, “More than once in your life” and “Multiple times.” The four items loaded to one underlying factor so an average of the four items was computed (Cronbach's alpha = 0.75).

Patient‐centered communication (PCC) was measured using the 6‐item scale adapted from the Consumer Assessment of Health Plans survey for use in the National Cancer Institute HINTS survey.^45^ Items map onto six dimensions of PCC, and ask how often in the past 12 months a healthcare professional did the following: “Give you the chance to ask all the health‐related questions you had”; “Give the attention you needed to your feelings and emotions?”; “Involve you in decisions about your healthcare as much as you wanted?”; “Make sure you understood the things you needed to do to take care of your health?”; “Help you deal with feelings of uncertainty about your health or healthcare?”; and “In the past 12 months, how often did you feel like you could rely on your doctors, nurses, or other health care professionals to take care of your health care needs?” Response options were between “Never” and “Always” on a 5‐point scale. These items loaded to one underlying factor with Cronbach's alpha of 0.93.

Perceived respect was measured using three items developed for this study, “in the last 12 months, how often did you feel your primary health care provider treated you...” “…with respect” and “…as an equal,” and “in the last 12 months, did you ever feel that your primary health care provider judged you because of your weight.” The first two items were measured on a 5‐point scale from “never” to “always” and the third item was measured as “yes” or “no”. The 5‐point scale items were rescaled to be bounded by 0 and 1 so that the items could be combined. The three items loaded to one factor and a mean score of the items was created (Cronbach's alpha = 0.70).

Delayed health care utilization was measured by a six‐item yes/no question scale.^34^ Respondents were asked if in the last 12 months they delayed or avoided getting health care because they…“gained weight”, “were told to lose weight”, “though you would be weighed”, “thought you would discuss weight”, “thought you would be asked to undress”, or “thought you could get rid of a problem by losing weight”. These items were collapsed into one variable representing a “yes” response to any item versus a “no” response to all items.

Doctor shopping was measured using a single yes/no item: “In the last 12 months, have you switched doctors or tried to find a new primary care doctor?”

BMI was measured by asking for the respondent's current height and weight without shoes on. Though BMI was available and used to identify the sample, the survey was sent anonymously, so current BMI was collected via the survey.

Patient demographic information was measured using standard survey items to assess age, race, ethnicity, education, and gender. Race was categorized into Black, White, and other race or ethnicity due to a small number of respondents who did not identify as White or Black. Education was categorized as high school graduate or less, some college, or a 4‐year college degree or more. Respondents were asked what their current weight and height without shoes was, and current BMI was calculated for each respondent. Finally, respondents were asked to indicate which of the following medical conditions they had ever been diagnosed with: arthritis, diabetes mellitus, high cholesterol, high blood pressure, heart disease, obstructive sleep apnea, depression, asthma, or other health problems. A variable represented a count of the number of these conditions indicated was created.

### 2.3 Analysis

In order to characterize the sample, summary statistics were calculated for all variables with findings presented using mean (SD) for continuous variables and frequency counts and percentages for nominal variables. Five multivariate linear or logistic regression analyses were used to assess the associations between BMI and the patient experience and patient behavior variables. Covariates included in these analyses included BMI, age, gender, race, education, and the number of comorbidities. Cases with missing data for variables included in a model were excluded for that model. The PROCESS macro for SPSS^46^ was used to fit the data to the conceptual path model and test the sequential and parallel mediation (PROCESS model 80) of the relationship between BMI and delayed health care utilization and doctor shopping by perceived stigma, PC communication, and perceived respect. Throughout this paper, the term “mediation” is used to describe an indirect effect of an independent variable on a dependent variable, and is not used to imply causation. Associations where delayed care or doctor shopping was the dependent variable are presented on the log odds scale. Pathway point estimates are the product of each association along the path, and path confidence intervals were calculated by bootstrapping based on selecting 5000 random samples of the data. All data are cross‐sectional, and thus this analysis is intended to test fit to a conceptual model, and not to suggest that these data demonstrate causality.

## 3 RESULTS

Sample characteristics are shown in Table 1. The majority of the sample (91.6%) identified as white and 61% identified as female gender. The average age was 59.3 years (SD = 14.4 years), with 3.4% under 30, 13.4% between 30 and 45, 28.6% between 46 and 59, 43.5% between 60 and 75, and 11.1% over 75. The average BMI in the sample was 35.1 kg/m^2^ (7.6 kg/m^2^).

**TABLE 1:** Sample characteristics, *n* = 2380 patients with BMI ≥ 25 from 5 of the 9 LHSNet sites

Variable	Missing % (*n*)	% (*n*) or mean (SD) among non‐missing
Age	0 (0)	59.3 (14.4)
Female gender	0 (0)	60.5 (1440)
Race	3.1 (73)	
White		91.6 (2114)
Black		2.9 (66)
Other		5.5 (127)
BMI	0 (0)	35.1 (7.6)
Education	1.8 (43)	
H.S. or less		20.9 (488)
Some college		36.5 (852)
College degree		42.7 (997)
Comorbidities	0 (0)	2.5 (1.8)
Site	0 (0)	
Site 1		17.8 (424)
Site 2		20.8 (494)
Site 3		21.0 (499)
Site 4		18.9 (451)
Site 5		21.5 (512)

Table 2 shows the distributions of the variables of interest and their bivariate associations with BMI. Delaying needed care and doctor shopping in the past year were reported by 27.5% and 13.7% respectively. All of the care quality and care utilization variables are associated with BMI in the hypothesized directions. Table 3 presents the results of multivariate linear and logistic regression models. BMI remains positively associated with frequency of experiencing stigmatizing situations (b = 0.03, *p* < 0.001), as well as a greater odds of having delayed care in the past year (OR = 1.06, *p* < 0.001) and having switched or attempted to switch primary doctors in the past year (OR = 1.02, *p* = 0.04). Modeling stigmatizing situations as a dichotomous variable (none vs. any) did not alter the pattern of results.

**TABLE 2:** Distributions of health care experience/utilization variables and bivariate associations with BMI in *n* = 2380 patients with BMI ≥ 25

Variable and scale range	Missing % (*n*)	Mean (SD) or % (*n*) among non‐missing	Bivariate association with BMI (B, *p*‐value)
Stigmatizing situations in health care (0–3)	5.3 (127)	0.57 (0.76)	0.04 (*p* < 0.001)
Patient‐centered communication (1–5)	5.5 (133)	3.5 (0.74)	−0.01 (*p* < 0.001)
Perceived respect (1–5)	4.8 (115)	0.91 (0.18)	−0.004 (*p* < 0.001)
Delayed needed care (yes/no)	3.7 (87)	27.5 (631)	0.07 (*p* < 0.001)
Doctor shopping (yes/no)	4.3 (103)	13.7 (313)	0.03 (*p* < 0.001)

**TABLE 3:** Multivariate regression model results for associations between BMI and health care experience and utilization variables in *n* = 2380 patients with BMI ≥ 25

Variable	Model 1: Stigma (B, *p*‐value)	Model 2: PC communication (B, *p*‐value)	Model 3: Respect (B, *p*‐value)	Model 4: Delayed care (OR, *p*‐value)	Model 5: Doctor shopping (OR, *p*‐value)
BMI	0.03 (<0.001)	−0.01 (<0.001)	−0.003 (<0.001)	1.06 (<0.001)	1.02 (0.049)
Age	−0.008 (<0.001)	0.003 (0.02)	0.001 (0.001)	0.99 (0.02)	0.98 (<0.001)
Female gender	0.06 (0.04)	−0.05 (0.15)	−0.01 (0.11)	0.67 (<0.001)	0.61 (0.001)
Race: White	−0.003 (0.97)	0.10 (0.13)	0.05 (0.002)	1.06 (0.79)	0.81 (0.40)
Race: Black	−0.22 (0.04)	−0.07 (0.56)	0.03 (0.34)	1.18 (0.64)	0.73 (0.47)
Race: Other	Ref	Ref	Ref	Ref	Ref
Education ≤ H.S. degree	−0.21 (<0.0001)	−0.03 (0.40)	−0.006 (0.58)	0.91 (0.52)	1.30 (0.12)
Education: Some college	−0.10 (0.004)	−0.03 (0.46)	0.001 (0.99)	0.86 (0.21)	1.02 (0.90)
Education ≥ college degree	Ref	Ref	Ref	Ref	Ref
Comorbidities	0.07 (<0.001)	0.01 (0.50)	−0.005 (0.05)	1.06 (0.09)	0.98 (0.58)
Site 1	−0.04 (0.40)	−0.006 (0.90)	−0.002 (0.05)	1.28 (0.11)	1.24 (0.29)
Site 2	−0.08 (0.10)	0.07 (0.17)	0.01 (0.23)	1.04 (0.80)	0.86 (0.47)
Site 3	−0.05 (0.29)	0.05 (0.36)	0.02 (0.16)	0.89 (0.46)	0.98 (0.94)
Site 4	−0.01 (0.90)	−0.03 (0.54)	−0.01 (0.33)	1.13 (0.42)	1.26 (0.25)
Site 5	Ref	Ref	Ref	Ref	Ref
*Intercept or constant*	−0.21 (0.09)	3.55 (<0.001)	0.91 (<0.001)	0.08 (<0.001)	0.42 (0.08)

**FIGURE 2:**
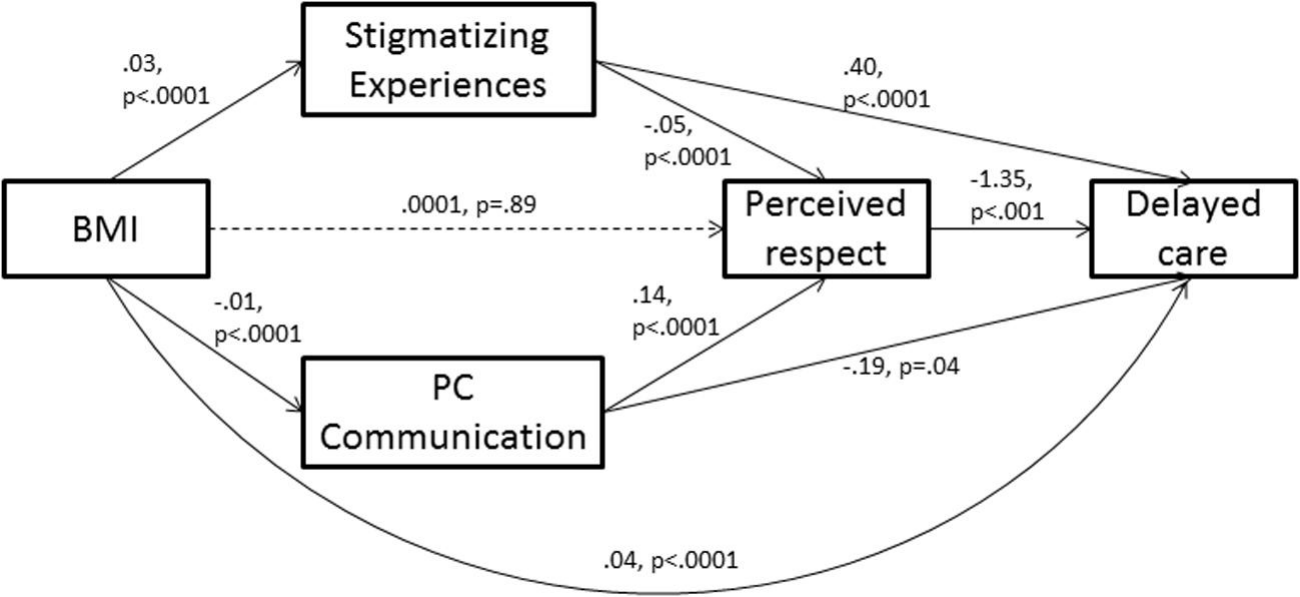
Path analysis model of delayed care utilization in *n* = 2,380 patients with BMI ≥ 25 (beta coefficients, *p*‐values shown)

**FIGURE 3:**
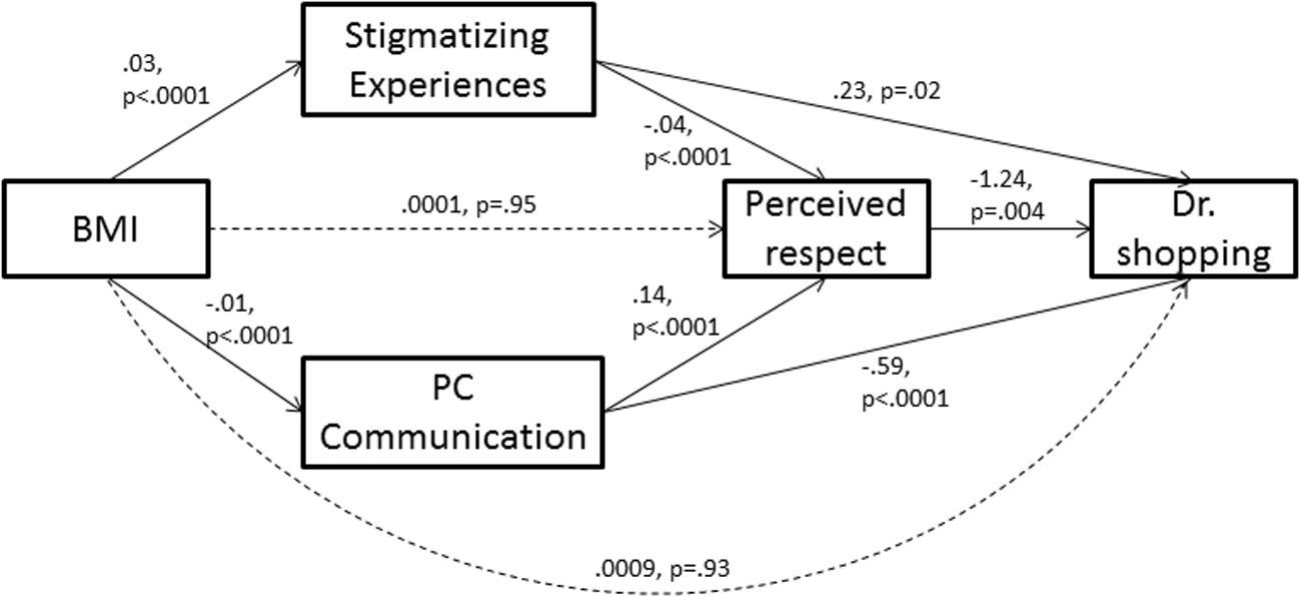
Path analysis model of doctor shopping in *n* = 2,380 patients with BMI ≥ 25 (beta coefficients, *p*‐values shown)

The path models with parameter estimates are shown in Figures 2 and 3. Beta coefficients and *p*‐values are presented for each path; solid lines represent significant associations and dotted lines represent non‐significant paths. Figure 1 shows that BMI is not directly associated with perceived respect when pathways through stigmatizing experiences and patient centered communication are modeled simultaneously. BMI is associated directly with delayed care (b = 0.04, 95% CI = 0.03, 0.06), and indirectly through more frequent stigmatizing experiences (indirect effect (b) = 0.01, (0.01, 0.03)), and less patient‐centered communication (b = 0.002 [0.0001, 0.005]). BMI is also associated with delaying care via a path through more frequent stigmatizing situations and lower perceived respect (b = 0.002 [0.001, 0.004]) and a path through less patient‐centered communication and lower perceived respect (b = 0.002 [0.001, 0.004]).

Figure 2 shows the pathways through which BMI is associated with doctor shopping. The direct effect of BMI on perceived respect (b = 0.0001, *p* = 0.95) and doctor shopping (b = 0.0009, *p* = 0.93) are no longer significant when modeled simultaneously with other mediators. However, all pathways through stigmatizing experiences and patient‐centered communication are significant. BMI is associated with doctor shopping indirectly through more frequent stigmatizing experiences (b = 0.01 [0.001, 0.01]), and less patient‐centered communication (b = 0.007 [0.003, 0.01]). BMI is also associated with doctor shopping via a path through more frequent stigmatizing situations and lower perceived respect (b = 0.002 [0.0004, 0.004]) and a path through less patient‐centered communication and lower perceived respect (b = 0.002 [0.0004, 0.004]).

## 4 DISCUSSION

The objective of this study was to test the pathways of a conceptual model of the association between BMI and health care delay or doctor shopping. The data were consistent with the hypothesized conceptual model. Among people with BMI greater than 24.9, higher BMI was associated with both delaying needed care and attempting to change primary doctors. BMI was also associated with two measures of interpersonal care quality: more frequent experiences of stigmatizing situations while receiving health care and less patient‐centered communication with health care providers. Both measures of interpersonal care quality mediated the association between BMI and each care utilization outcome. BMI was also associated with less perceived respect from health care providers. The association between BMI and perceived respect was mediated by reported experiences of both stigmatizing situations and patient‐centered communication. Perceived respect is in turn associated with delaying needed care and attempting to change doctors.

One may interpret these associations as evidence that patients with higher BMI feel less respected due to their experiences of stigma and the lower quality communication they receive. After experiencing a stigmatizing situation or poor communication quality, a patient may delay care or seek an alternative source of care, either due to the perceived lack of respect, or independent of perceived respect. The direct unmediated associations between stigmatizing experiences/lower quality communication and care delay/doctor shopping may be the result of the patient perception of lower quality interpersonal care or perhaps resulting from patients wanting to avoid embarrassment from being unable to lose weight as recommended. It is also a possibility that patients who experience identity threat in an encounter avoid follow‐up encounters to avoid the stress that accompanies those experiences. Since identity threat is partially an unconscious process, the patient may not recall feeling disrespected, only that something made them uncomfortable.

These findings are consistent with evidence that people with higher BMIs are more likely to feel judged and discriminated against by their health care providers,^13^, ^25^, ^26^, ^47^ and evidence that physicians and other health care providers engage in less rapport building, offer less education, and spend less time overall with patients with higher BMIs.^12^, ^13^, ^14^, ^15^, ^16^, ^48^ The findings also support research that has found that health care providers report having less respect for their patients with obesity.^9^, ^26^ The findings extend this work to show that these experiences may contribute to avoiding or delaying care and seeking a new doctor in this patient population. In addition, these findings are consistent with the hypotheses that people with obesity are more likely to have an unsatisfactory health care experience, and may experience identity threat due to perceived stigmatization and, thus, are more likely to avoid seeing that same doctor again. After modeling all mediation pathways simultaneously, the direct effect of BMI on delayed care remains significant, suggesting there are additional unmeasured mediators of the relationship; whereas the direct association between BMI and doctor shopping in no longer significant, suggesting that there are not additional indirect effects and the mediators in the model explain the association between BMI and doctor shopping.

This study was limited to individuals with BMIs corresponding to overweight or obesity only. Thus, the results may be different if the sample included people from the normal weight BMI category, who may be less likely to experience discrimination related to their body size. Results may be more conservative than if they included people from this nonstigmatized group.

Limitations of this study include the cross‐sectional study design. While conclusions about causality in the observed associations are not appropriate, the data do fit the conceptual model as hypothesized. Nonetheless, it is important not to assume that causality is demonstrated by these data, just that they are consistent with the hypothesized causal path model. These relationships should be examined in a more rigorous longitudinal study design. Additionally, a response rate maximization strategy was not used, and the survey response rate was lower than ideal (14.2%). The stratified sampling scheme provided representativeness across the overweight and obesity BMI spectrum; although respondents had, on average, lower BMI's than non‐responders. Additionally, the sample was disproportionally white compared to the U.S., and generalizability should be considered with that in mind. There is also no way to know whether responding to the questionnaire was associated with the dependent or mediating variables. Finally, the measure of stigmatizing situations yielded a low prevalence of these experiences. This a common problem with measurement in this area of research, and it should be acknowledged that there are other types of discrimination experiences or microaggressions that are not picked up by this measure.

Despite these limitations, this study provides evidence for a conceptual model of how perceived stigma and poor communication, two factors related to health care provider attitudes and beliefs about people with obesity, contribute to care avoidance and delay in patients with obesity. This model may be useful for designing intervention strategies to improve care utilization and subsequent outcomes in patients living with overweight or obesity. For example, health care organizations might consider focusing on the care experiences of people with overweight or obesity in order to improve utilization of preventive care appointments or recommended testing in patients managing chronic diseases like type 2 diabetes. Strategies to reduce the frequency of stigmatizing experiences and improve patient‐centered communication with patients with obesity might include educating clinical staff on appropriate and patient‐preferred strategies for discussing weight, or on non‐behavioral contributors to obesity, strategies shown to reduce weight bias. They might also focus on developing and practicing skills in partnership‐building, patient‐centered communication strategies like motivational interviewing, or perspective taking to improve empathy for the situations and experiences of diverse patients.

## 5 CONCLUSION

These results are consistent with a model suggesting that the association between BMI and health care avoidance are mediated by stigmatizing situations in medical care settings, poor patient‐provider communication, and patient perception that they are not respected by their health care provider. People who experience stigma may thus delay care for new symptoms because they feel uncomfortable seeing their doctor, or do not have a trusted primary care doctor to see. As a result, they may present later in the progression of a disease and face a worse prognosis. Interventions to improve health care provider communication about weight and with people with obesity may be called for to reduce negative experiences and subsequent care delay.

## CONFLICT OF INTEREST

The authors declare no conflict of interest.

## AUTHOR CONTRIBUTIONS

Sean M. Phelan led the development and conceptualization of the manuscript and drafted it. All authors contributed important conceptual information and critically reviewed the manuscript. Sean M. Phelan, Katherine W. Bauer, David Bradley, Steven M. Bradley, Irina V. Haller, Manpreet Mundi, Lila J. Finney Rutten, and Ivana Croghan contributed to the development of the survey and collected the data at their respective institutions. Ivana Croghan was the principal investigator of the overall study. Darrel Schroeder and Kristin Fischer conducted all regression analyses, and Kristin Fischer and Sean M. Phelan conducted indirect effects analyses.

## References

[1] Alberga AS , Russell‐Mayhew S , von Ranson KM , McLaren L . Weight bias: a call to action. J Eat Disord. 2016;4:34. 27826445 10.1186/s40337-016-0112-4 PMC5100338

[2] Phelan SM , Burgess DJ , Yeazel MW , Hellerstedt WL , Griffin JM , van Ryn M . Impact of weight bias and stigma on quality of care and outcomes for patients with obesity. Obes Rev. 2015;16(4):319‐326. 25752756 10.1111/obr.12266 PMC4381543

[3] Phelan SM , Dovidio JF , Puhl RM , et al. Implicit and explicit weight bias in a national sample of 4,732 medical students: the medical student CHANGES study. Obes. 2013;11(9). 10.1002/oby.20687 PMC3968216 24375989

[4] Schwartz M , Chambliss H , Brownell K , Blair S , Billington C . Weight bias among health professionals specializing in obesity. Obes Res. 2003;11(9):1033‐1039. 12972672 10.1038/oby.2003.142

[5] Puhl RM , Himmelstein MS , Pearl RL . Weight stigma as a psychosocial contributor to obesity. Am Psychol. 2020;75(2):274‐289. 32053000 10.1037/amp0000538

[6] Tomiyama AJ , Finch LE , Belsky ACI, et al. Weight bias in 2001 versus 2013: contradictory attitudes among obesity researchers and health professionals. Obes. 2015;23(1):46‐53. 10.1002/oby.20910 25294247

[7] Puhl RM , Heuer CA . The stigma of obesity: a review and update. Obes. 2009;17(5):941‐964. 10.1038/oby.2008.636 19165161

[8] Foster GD , Wadden TA , Makris AP , et al. Primary care physicians' attitudes about obesity and its treatment. Obes Res. 2003;11(10):1168‐1177. 14569041 10.1038/oby.2003.161

[9] Hebl MR , Xu J . Weighing the care: physicians' reactions to the size of a patient. Int J Obes Relat Metab Disord. 2001;25(8):1246‐1252. 11477511 10.1038/sj.ijo.0801681

[10] Huizinga MM , Bleich SN , Beach MC , Clark JM , Cooper LA . Disparity in physician perception of patients' adherence to medications by obesity status. Obes. 2010;18(10):1932‐1937. 10.1038/oby.2010.35 PMC3149807 20186132

[11] Persky S , Eccleston CP . Medical student bias and care recommendations for an obese versus non‐obese virtual patient. Int J Obes. 2010;35(5):728‐735. 10.1038/ijo.2010.173 PMC3000449 20820169

[12] Phelan SM , Lynch B , Blake KD , et al. The impact of obesity on patient‐centered communication. Obes Sci Pract. 2018;4(4):338‐346. 30151228 10.1002/osp4.276 PMC6105704

[13] Richard P , Ferguson C , Lara AS , Leonard J , Younis M . Disparities in physician‐patient communication by obesity status. Inquiry. 2014;51. 10.1177/0046958014557012 PMC5813620 25432989

[14] Wong MS , Gudzune KA , Bleich SN . Provider communication quality: influence of patients' weight and race. Patient Educ Counsel. 2015;98(4):492‐498. 10.1016/j.pec.2014.12.007 PMC4379992 25617907

[15] Gudzune KA , Beach MC , Roter DL , Cooper LA . Physicians build less rapport with obese patients. Obes. 2013;21(10):2146‐2152. 10.1002/oby.20384 PMC3694993 23512862

[16] Bertakis KD , Azari R . The impact of obesity on primary care visits. Obes Res. 2005;13(9):1615‐1623. 16222065 10.1038/oby.2005.198

[17] Wanzer MB , Booth‐Butterfield M , Gruber K . Perceptions of health care providers' communication: relationships between patient‐centered communication and satisfaction. Health Commun 2004;16(3):363‐384. 15265756 10.1207/S15327027HC1603_6

[18] Zolnierek KB , Dimatteo MR . Physician communication and patient adherence to treatment: a meta‐analysis. Med Care. 2009;47(8):826‐834. 19584762 10.1097/MLR.0b013e31819a5acc PMC2728700

[19] Bertakis KD , Azari R . Patient‐centered care is associated with decreased health care utilization. J Am Board Fam Med. 2011;24(3):229‐239. 21551394 10.3122/jabfm.2011.03.100170

[20] Bertakis KD , Azari R . Determinants and outcomes of patient‐centered care. Patient Educ Counsel. 2011;85(1):46‐52. 10.1016/j.pec.2010.08.001 20801601

[21] Stewart M , Brown JB , Donner A , et al. The impact of patient‐centered care on outcomes. J Fam Pract. 2000;49(9):796‐804. 11032203

[22] Pollak KI , Alexander SC , Coffman CJ , et al. Physician communication techniques and weight loss in adults: project CHAT. Am J Prev Med. 2010;39(4):321‐328. 20837282 10.1016/j.amepre.2010.06.005 PMC2939864

[23] Pollak KI , Alexander SC , Ostbye T , et al. Primary care physicians' discussions of weight‐related topics with overweight and obese adolescents: results from the Teen CHAT Pilot study. J Adolesc health official publication of the Society for Adolescent Medicine. 2009:45(2):205‐207. 19628149 10.1016/j.jadohealth.2009.01.002 PMC2753373

[24] Myers A , Rosen JC . Obesity stigmatization and coping: relation to mental health symptoms, body image, and self‐esteem. Int J Obes Relat Metab Disord. 1999;23(3):221‐230. 10193866 10.1038/sj.ijo.0800765

[25] Gudzune KA , Bennett WL , Cooper LA , Bleich SN . Patients who feel judged about their weight have lower trust in their primary care providers. Patient Educ Counsel. 2014;97(1). 10.1016/j.pec.2014.06.019 PMC4162829 25049164

[26] Huizinga MM , Cooper LA , Bleich SN , Clark JM , Beach MC . Physician respect for patients with obesity. J Gen Intern Med. 2009;24(11):1236‐1239. 19763700 10.1007/s11606-009-1104-8 PMC2771236

[27] Branscombe NR , Ellemers N , Spears R , Doosje B . The context and content of social identity threat. In: Ellemers N , Spears R , Doosje B , eds. Social identity: Context, Commitment, Content. Oxford: Blackwell; 1999:35‐55.

[28] Major B , O'Brien LT . The social psychology of stigma. Annu Rev Psychol. 2005;56:393‐421. 15709941 10.1146/annurev.psych.56.091103.070137

[29] Steele CM . A threat in the air. How stereotypes shape intellectual identity and performance. Am Psychol. 1997;52(6):613‐629. 9174398 10.1037//0003-066x.52.6.613

[30] Brändstedt J , Wangefjord S , Nodin B , Gaber A , Manjer J , Jirström K . Gender, anthropometric factors and risk of colorectal cancer with particular reference to tumour location and TNM stage: a cohort study. Biol Sex Differ. 2012;3(1):23. 23072404 10.1186/2042-6410-3-23 PMC3504577

[31] Berrington de Gonzalez A , Hartge P , Cerhan JR , et al. Body‐mass index and mortality among 1.46 million white adults. N. Engl J Med. 2010;363(23):2211‐2219. 21121834 10.1056/NEJMoa1000367 PMC3066051

[32] Sutin AR , Stephan Y , Terracciano A . Weight discrimination and risk of mortality. Psychol Sci. 2015;26(11):1803‐1811. 26420442 10.1177/0956797615601103 PMC4636946

[33] Fontaine KR , Faith MS , Allison DB , Cheskin LJ . Body weight and health care among women in the general population. Arch Fam Med. 1998;7(4):381‐384. 9682694 10.1001/archfami.7.4.381

[34] Drury CA , Louis M . Exploring the association between body weight, stigma of obesity, and health care avoidance. J Am Acad Nurse Pract. 2002;14(12):554‐561. 12567923 10.1111/j.1745-7599.2002.tb00089.x

[35] Gudzune KA , Bennett WL , Cooper LA , Clark JM , Bleich SN . Prior doctor shopping resulting from differential treatment correlates with differences in current patient‐provider relationships. Obes. 2014;22(9). 10.1002/oby.20808 PMC4149586 24942593

[36] Gudzune KA , Bleich SN , Richards TM , Weiner JP , Hodges K , Clark JM . Doctor shopping by overweight and obese patients is associated with increased healthcare utilization. Obes. 2013;21(7):1328‐1334. 10.1002/oby.20189 PMC3742565 23671015

[37] Van Walraven C , Oake N , Jennings A , Forster AJ . The association between continuity of care and outcomes: a systematic and critical review. J Eval Clin Pract. 2010;16(5):947‐956. 20553366 10.1111/j.1365-2753.2009.01235.x

[38] Lambrew JM , DeFriese GH , Carey TS , Ricketts TC , Biddle AK . The effects of having a regular doctor on access to primary care. Med Care. 1996;34(2):138‐151. 8632688 10.1097/00005650-199602000-00006

[39] Villaseñor S , Krouse HJ . Can the use of urgent care clinics improve access to care without undermining continuity in primary care? J Am Assoc Nurse Pract. 2016;28(6):335‐341. 26485113 10.1002/2327-6924.12314

[40] Ellemers N , Spears R , Doosje B . Self and social identity. Annu Rev Psychol. 2002;53(1):161‐186. 11752483 10.1146/annurev.psych.53.100901.135228

[41] Schmader T , Johns M . Converging evidence that stereotype threat reduces working memory capacity. J Pers Soc Psychol. 2003;85(3):440‐452. 14498781 10.1037/0022-3514.85.3.440

[42] Croghan IT , Phelan SM , Bradley DP , et al. Needs assessment for weight management: the learning health system network experience. Mayo Clin Proc Innov Qual Outcomes. 2018;2(4):324‐335. 30560234 10.1016/j.mayocpiqo.2018.08.001 PMC6260476

[43] Finney Rutten L , Alexander A , Embi P , et al. Patient‐centered network of learning health systems: developing a resource for clinical translational research. J Clin Transl Sci. 2017;1(1):40‐44. 28515960 10.1017/cts.2016.11 PMC5413962

[44] DeJesus R , Bauer K , Bradley D , et al. Experience and expectations of patients on weight loss: the learning health system network experience. Obes Sci Pract. 2019;5(5):479‐486. 31687172 10.1002/osp4.364 PMC6820006

[45] Underhill ML , Kiviniemi MT . The association of perceived provider‐patient communication and relationship quality with colorectal cancer screening. Health Educ Behav. 2012;39(5):555‐563. 21986241 10.1177/1090198111421800 PMC3627480

[46] Hayes AF . Introduction to Mediation, Moderation, and Conditional Process Analysis: A Regression‐Based Approach. 2^nd^ ed. New York: Guilford Press; 2017.

[47] Gudzune KA . Perceived judgment about weight can negatively influence weight loss: a cross‐sectional study of overweight and obese patients. Prev Med. 2014;62:103‐107. 24521530 10.1016/j.ypmed.2014.02.001 PMC4006987

[48] Tilburt JC , Byrne TO , Branda ME , Phelan S . Higher BMI associated with shorter visits in male oncology patients: an exploratory analysis. Patient Educ Counsel. 2019;102(12):2353‐2357. 10.1016/j.pec.2019.07.009 PMC6851463 31331706

